# What are the advantages of living in a community? A microbial biofilm perspective!

**DOI:** 10.1590/0074-02760180212

**Published:** 2018-07-26

**Authors:** André Luis Souza dos Santos, Anna Clara Milesi Galdino, Thaís Pereira de Mello, Lívia de Souza Ramos, Marta Helena Branquinha, Ana Maria Bolognese, José Columbano, Maryam Roudbary

**Affiliations:** 1 Universidade Federal do Rio de Janeiro, Instituto de Microbiologia Paulo de Góes, Departamento de Microbiologia Geral, Laboratório de Estudos Avançados em Microrganismos Emergentes e Resistentes, Rio de Janeiro, RJ, Brasil; 2 Universidade Federal do Rio de Janeiro, Instituto de Química, Programa de Pós-Graduação em Bioquímica, Rio de Janeiro, RJ, Brasil; 3 Universidade Federal do Rio de Janeiro, Faculdade de Odontologia, Departamento de Odontopediatria e Ortodontia, Rio de Janeiro, RJ, Brasil; 4 Faculdades São José, Faculdade de Odontologia, Disciplina de Ortodontia e Clínica Integrada Infantil, Rio de Janeiro, RJ, Brasil; 5 Iran University of Medical Sciences, School of Medicine, Department of Medical Mycology and Parasitology, Tehran, Iran

**Keywords:** biofilm, microbial lifestyle, virulence, resistance, tolerance, anti-biofilm strategies

## Abstract

Biofilm formation is the preferred mode of growth lifestyle for many microorganisms, including bacterial and fungal human pathogens. Biofilm is a strong and dynamic structure that confers a broad range of advantages to its members, such as adhesion/cohesion capabilities, mechanical properties, nutritional sources, metabolite exchange platform, cellular communication, protection and resistance to drugs (e.g., antimicrobials, antiseptics, and disinfectants), environmental stresses (e.g., dehydration and ultraviolet light), host immune attacks (e.g., antibodies, complement system, antimicrobial peptides, and phagocytes), and shear forces. Microbial biofilms cause problems in the hospital environment, generating high healthcare costs and prolonged patient stay, which can result in further secondary microbial infections and various health complications. Consequently, both public and private investments must be made to ensure better patient management, as well as to find novel therapeutic strategies to circumvent the resistance and resilience profiles arising from biofilm-associated microbial infections. In this work, we present a general overview of microbial biofilm formation and its relevance within the biomedical context.

*The social life of microorganisms* - Microorganisms can colonise virtually every environment on Earth, including soils, water, and air-liquid interfaces - each of which present distinct physicochemical conditions. The ability to quickly adapt to different habitats can be explained, at least in part, by the fact that microbial cells are the most ancient representative lineage of living organisms and they have experienced many changes in environment over their billions of years of existence. This evolution has permitted the development of plastic genomes and, consequently, plastic metabolisms in many microorganisms, which allows for rapid mutation (plastic response) when faced with adversity.^1^ With this perception in mind, curiously, microorganisms have been developing an amazing ability to resist diverse, and sometimes drastic, environmental insults and stresses. They have learned to live together in an “organised and well-orchestrated community” - the so-called “biofilm”.

The word “*community*” is derived from the Old French “*comuneté*”, which comes from the Latin “*communis*”, meaning “shared in common”. Community can be defined as a social group (an assemblage of interacting populations) of any size, whose members occupy a given area or a specific locality, share common characteristics or interests, establish communication platforms, and often have a common heritage. In the microbial world, the concept of living together can be applied to all of these avenues, contemplating the integration and fulfilment of all of the needs of a group. Furthermore, living together stimulates and promotes several beneficial features for microorganisms compared to living a solitary life. Undoubtedly, protection and tolerance/resistance are the most beneficial aspects of being an active participant within a well-established microbial community.^2^

*Biofilm: the preferred microbial lifestyle* - The idea that microorganisms are able to live together and form biofilms is indeed an old one, dating back to the classical and primordial studies by Antonie van Leeuwenhoek (1632-1723), who first reported the concept of “microbial aggregation” on the surface of teeth, and Louis Pasteur (1822-1895), who described the microbial community to be the cause of wine becoming acetic.^3^ Generally, biofilms are defined as communities of properly organised microorganisms (as a typical social cooperation system) attached to an inert or living substrate (Fig. 1) and embedded in a self-produced extracellular matrix (also called extracellular polymeric substance) composed of (glyco)proteins, (glyco)lipids, (mono)/(poly)saccharides, extracellular DNA, minerals, and water, which works like an adhesive favouring cell-cell and cell-substrate interactions. Additionally, the biofilm extracellular matrix can contain host-derived components, such as human serum, saliva glycoproteins, and vaginal excretions.^4^ ^-^ ^8^

The chemical constituents of the extracellular matrix are responsible for maintaining biofilm architecture, stabilising it through the formation of intermolecular interactions, cross-linkages of multivalent cations, and an ingrained network of biopolymers. All these extracellular matrix properties help to form a robust shelter that offers a protected and nutritionally rich ecological niche, contributing to microbial survival, molecule exchanges, communication (by *quorum sensing* signalling, a cell density-dependent communication system that regulates cooperative behaviours), and proliferation. The extracellular matrix is an extremely dynamic structure that is constantly remodelled to suit the environment and living conditions of the cells, as well as to provide physicochemical stability to the biofilm architecture.^4^ ^-^ ^8^

**Fig. 1:**
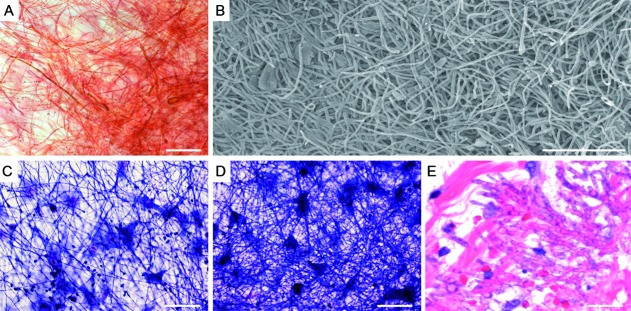
biofilm formation by the filamentous fungus, *Scedosporium apiospermum*, an opportunistic human pathogen, on both abiotic and biotic surfaces. The biofilm on the polystyrene substrate was detected by safranin staining (A) and by scanning electron microscopy (B). The co-culture of *S. apiospermum* conidia with lung epithelial A549 cells for 24 h (C) and 48 h (D) revealed a dense mycelial mass (a typical biofilm structure) covering the lung epithelial cells, as evidenced by Giemsa staining. Histopathological section stained by hematoxylin and eosin showing many hyphae in the skin biopsy from human infected tissue (E). Bars, 50 µm. For more detail see Mello et al.^7^ Image credit: Fig. E was kindly gifted by Dr Stacy Beal, Assistant Professor, University of Florida, College of Medicine, Department of Pathology, Immunology and Laboratory Medicine, USA.

Biofilm formation is a sequential, finely orchestrated, multi-step phenomenon governed by a number of physical, chemical, and biological factors: attachment, microcolony formation (proliferation), maturation, and dispersion (desorption/detachment) (Fig. 2).^8^ ^-^ ^10^ During these phases, microbial biofilms form three-dimensional (3D) structures that are separated by water channels, which allow the entry of nutrients and oxygen, as well as the discharge of waste products. Moreover, the creation of a myriad of microenvironments within the biofilm community allows for complex microbial interactions, including chemical and electrochemical cell­-to­-cell communication and enhanced horizontal gene transfer.^6^ ^,^ ^11^ In the clinical context, horizontal gene transfer can determine, for instance, the acquisition of antimicrobial resistance genes, a phenomenon well-known in bacteria. The presence of environmental DNA (eDNA; which is a complex mixture of cellular genomic DNA from living organisms within a population and coming from autolysis, uncontrolled cell lysis or active secretion systems via membrane vesicles), and extracellular DNA (e.g., coming from viruses) in a biofilm is widespread and constitutes an evolutionary advantage. eDNA plays many relevant roles, including as a structural molecule for biofilm stability, as a source of energy and nutrients (carbon, nitrogen, and phosphorus), and as a means of horizontal gene transfer by transformation of competent sister bacteria.^12^

It is important to emphasise that biofilms have varied structures, 3D architectures, and chemical compositions, and that these factors are influenced by the particular microbial genera, species, and strains, as well as by the environmental conditions.^4^ ^-^ ^6^ ^,^ ^8^ ^,^ ^11^ ^,^ ^13^ ^-^ ^15^ Interestingly, exposing the microorganism to sub-inhibitory concentrations of classical antimicrobial drugs can induce biofilm formation and the expression of additional virulence attributes. For instance, the administration of sub-inhibitory doses of aminoglycosides was able to trigger biofilm formation in the bacterial pathogens *Escherichia coli* and *Pseudomonas aeruginosa*.^16^ Similarly, mature biofilms of *Candida albicans*, when challenged with sub-minimum inhibitory concentrations (MICs) of fluconazole, were able to secrete higher quantities of aspartic peptidases (Saps), a well-recognised multitask virulence factor, compared to untreated biofilms.^17^ Undoubtedly, biofilm formation is an extremely advantageous outcome for microbial survival, development, and evolution.

*Microbial biofilm: a real problem in the clinical setting* - The incidence of biofilm-associated infections is increasing annually, and their impact on health and services may be grossly underestimated. The National Institutes of Health estimated that both bacterial and fungal biofilms were responsible for over 80% of all nosocomial infections in the USA.^18^ Biofilm-related illnesses can be divided into three main types: (i) device-related biofilm disease, (ii) non-device-related chronic biofilm disease, and (iii) biofilm-related device malfunction.^19^ Biofilm-associated infections represent a major problem in the hospital setting worldwide due to their recalcitrant nature and to difficulties in treatment.^9^ ^,^ ^10^ ^,^ ^20^ ^,^ ^21^ Microbial infections related to biofilms are usually treated with high doses of antimicrobials (usually a combination of different drugs) for a prolonged period of time.^9^ ^,^ ^22^ ^-^ ^24^ Such a treatment regimen can provoke unpleasant collateral consequences for the patient, and in some cases will involve the “traumatic” (with a significant physical and psychological burden) replacement or removal of medical implants.^9^ Consequently, biofilm formation is a major contributor to the unacceptably high mortality rates associated with microbial infections, particularly those caused by bacterial and fungal pathogens.^19^ ^,^ ^22^ Corroborating this statement, more than 500,000 deaths per year occur due to biofilm-related infections in the USA.^25^

The biofilm lifestyle is associated with the chronic nature of microbial infections (Table I),^26^ ^-^ ^34^ particularly in hospitalised patients under care with a medical device (Table II),^35^ resulting in prolonged hospital stay and added healthcare costs to both public and private institutions worldwide.^9^ ^,^ ^10^ ^,^ ^18^ ^-^ ^24^ It has been estimated that, for hospitals in the USA alone, the annual cost for treating device-related biofilm infections is over eleven billion dollars.^36^ To compound this problem, medical devices and tissue engineering constructs are susceptible to colonisation by different microbial groups and can induce (i) device malfunction, (ii) chemical degradation of biomaterials, and/or (iii) infectious processes by different classes of microorganisms.^37^

In addition, biofilms can serve as a source of local and systemic infections, especially in patients confined in intensive care units. For instance, biofilm formation by *Candida* spp. in the respiratory tract of gravely ill patients (i.e., immunocompromised patients) can promote the development of ventilator-associated bacterial pneumonia by *P. aeruginosa* and *Staphylococcus aureus*.^38^ A single microbial species or a consortia of multiple species can cohabitate within a mature biofilm structure. Polymicrobial biofilms are considered to be even more complex communities of microorganisms that cooperatively interact in an altruistic manner, and are commonly observed in natural environments and in *in vivo* infections (Fig. 3), imposing additional complications in the management and treatment of diseases.^39^ ^,^ ^40^ Dental plaque is a typical polymicrobial biofilm (main cariogenic bacteria are streptococci, actinomycetes, and lactobacilli) that forms on the surfaces of teeth and, if inadequately controlled, can lead to dental caries (which can lead to tooth decay) or periodontitis.^41^ Another relevant issue when considering polymicrobial biofilms is the variable and metabolic-rich activities in this closed community that favours cooperation (which can be comparable to that in the tissues of multicellular organisms) and protection (e.g., the presence of catalase-producing microorganisms that will protect non-catalase-producing species from the detrimental effects of hydrogen peroxide).^42^

Biofilm formation is considered a virulence determinant in microorganisms, and it strongly contributes to microbial resistance to conventional antimicrobial agents, host protective immune responses, hostile environmental pressures/stresses, predation, and shear forces.^43^ ^-^ ^47^ Interestingly, dispersed biofilm microbial cells have a greater capability to cause cytotoxicity, virulence, and mortality than their planktonic counterparts.^48^ The biofilm tolerance/resistance phenomenon is associated with (i) a high density of microbial cells forming the mature biofilm structure, which presents distinct growth and metabolic rates, (ii) the low penetration of drugs through the extracellular matrix and/or covalent binding of antimicrobial drugs to the extracellular matrix components, (iii) the differential expression (down or upregulation) of drug targets, (iv) the efflux of intracellular drugs through pumps and transporter proteins, (v) the upregulation of different classes of resistance genes and alteration in the cellular stress responses, and (vi) the presence of a small and distinct subpopulation of microbial cells called ‘persisters’ that is spontaneously formed within a biofilm. Persisters are defined as dormant, metabolically inactive and non-dividing variants of regular cells and are highly recalcitrant to antimicrobial challenge.^43^ ^-^ ^47^ Indeed, the mechanisms underlying biofilm resilience are complex and multifactorial; in this way, one or many of these previously proposed phenomena may be operational and contribute to the broad range of resistance against multiple stressors.

**Fig. 2:**
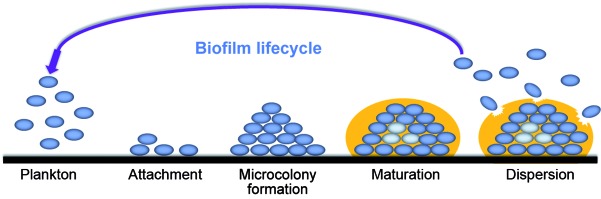
biofilm development steps produced by microorganisms. The image indicates the adherence of a microbial community to the surface, followed by microcolony formation, the maturation of the biofilm 3D architecture (note the presence of different cells, including the persisters, which are represented by light blue circles) with the presence of an extracellular polymeric matrix (represented by an orange cover) and, finally, its dispersion, which can lead to the colonisation and formation of a new biofilm structure in a distinct place, as well as the persistence of the infectious disease.

**TABLE I:** Human diseases associated with biofilm development

Chronic prostatitis	Orthopedic infections (e.g., osteomyelitis)
Chronic wounds	Otitis media
Chronic lung infection in cystic fibrosis patients	Periodontal disease (e.g., gingivitis)
Dental caries	Tuberculosis
Endocarditis	Upper respiratory infections (e.g., rhinosinusitis)
Keratitis	Urinary tract infections

**TABLE II:** Medical devices and implants in which microbial biofilm formation is a problem

Abdominal drains	Intrauterine contraceptive devices
Artificial voice prostheses	Intravenous catheters
Artificial hip prostheses	Nephrostomy tubes
Breast implants	Orthodental prosthetics
Cannulation	Orthopedic devices
Cardiac pacemakers	Peritoneal dialysis catheters
Cardiovascular valves	Prosthetic joints
Cerebrospinal fluid shunts	Silicone rubber prosthesis
Contact or intraocular lenses	Stents
Dental implants	Tissue fillers
Electrical dialyzers	Urinary catheters
Endotracheal tubes	Ventriculoperitoneal shunts
Implanted prosthetic devices for erectile dysfunction	Voice prostheses

**Fig. 3:**
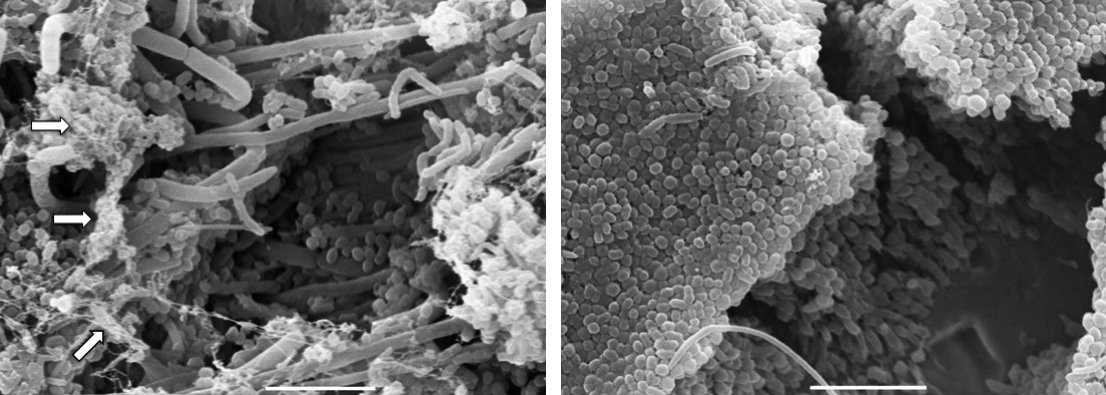
polymicrobial biofilms detected on orthodontic appliances visualised by scanning electron microscopy (SEM). The SEM images reveal the presence of different microbial groups, including bacteria and fungi, and an extracellular matrix (arrows). Bars, 5 µm.

The relevance of the microbial biofilm for numerous biotechnological and biomedical areas is reflected, at least in part, by the significant increase in the number of publications about biofilms over recent years, confirming that it is a current and vigorous research field. Herein, we have assimilated data gleaned from the available literature, in which the search was performed on April 1st, 2018, using the PubMed database (www.pubmed.com). To accomplish this, the keyword “biofilm” was added to the “Advanced Search Builder tool” and only papers published in English containing these words in the title/abstract were selected. In addition, the list of results was exported to the EndNote software in order to eliminate duplicated references. Our results revealed a total of 30,453 publications (research papers, short communications, (mini)reviews, and book chapters) concerning studies on biofilms during the period 1980-2017 (Fig. 4A). A meticulous data analysis indicated that the number of publications increased significantly when the periods 1990-1999 (≈ 612%), 2000-2009 (≈ 4435%), and 2010-2017 (≈ 12550%) were compared to the period 1980-1989 (Fig. 4B).

Future efforts should explore the integration of diverse methods employed to successfully combat microbial biofilm formation, which has become a severe problem worldwide. In this context, biofilm inhibition and destruction represent high value targets for the development of new antimicrobial drugs and/or new anti-biofilm strategies (Fig. 5), particularly against the extracellular matrix, which acts as a sponge.^49^ Two major approaches can be taken into account to combat biofilm-related infections: obstruction of biofilm formation (e.g., by modifying the physicochemical properties of inert surfaces through coating or impregnating surfaces with antimicrobial compounds, or by targeting specific surface microbial adhesins using lectins or antibodies), and disarticulation of the mature biofilm (e.g., by destabilising, weakening, or destroying the extracellular matrix components using hydrolytic enzymes such as DNases, glycosidases, lipases, and proteases; surfactants; chelator agents; biocides; or inhibitors of *quorum sensing* systems).^34^ ^,^ ^49^ ^,^ ^50^ With these ideas in mind, it is likely that an effective anti-biofilm strategy will emerge from a fluid and dynamic cross-talk involving distinct biomedical disciplines.

**Fig. 4:**
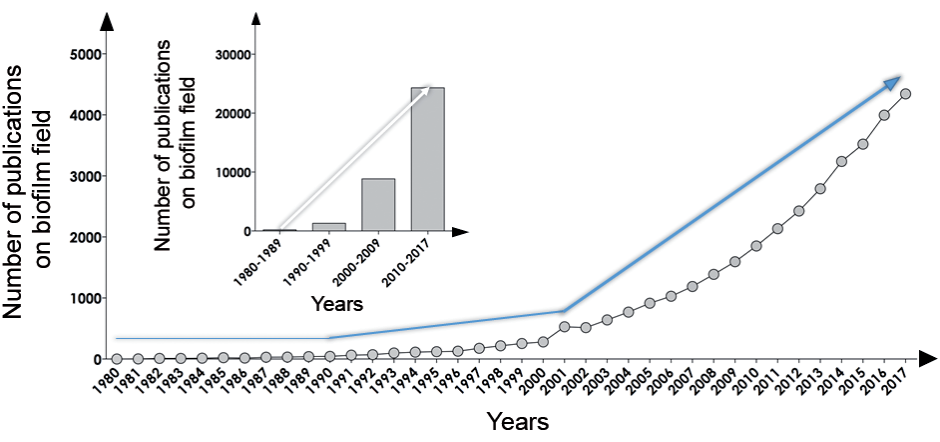
biofilm publications. The search was performed in the PubMed database (www.pubmed.com) on April 1st, 2018, using the keyword “biofilm”, and only papers published in English containing this word in the title and/or in the abstract were selected for analysis. Subsequently, the list of results was exported to the EndNote software (version X1), using the “Output Records” tool in order to eliminate duplicated references by means of the “Find Duplicates” tool. The graphic shows the absolute number of publications in the biofilm field during the period 1980-2017 (blue lines denote the increasing number of publications over the years). Note the substantial increase in the number of publications across the timeline, especially comparing the period of 1980-1989 with subsequent periods.

An additional relevant issue that must be highlighted is the fact that the currently used antimicrobial agents in the clinical arena were developed to act on planktonic cells. As such, these prescription drugs are essentially only effective against microbial cells during their exponential growth phase, and consequently, are either poorly effective or totally ineffective against cells in a biofilm. For example, biofilm-forming microbial cells can have from 10 to > 1,000-fold greater minimum inhibitory concentration of classical antimicrobial drugs compared to their planktonic counterparts when treated under laboratory conditions.^7^ ^,^ ^51^ All of these facts emphasise the necessity to perform susceptibility testing on new drug formulations using biofilm-forming microbial cells as a routine in both research labs and hospital settings, as well as in clinical trials. In order to reach this utopic, ideal, simple, cheap, rapid, reproducible, rigorous, and responsive *in vitro* test should be standardised and validated. Furthermore, its efficacy should be proven to aid in predicting *in vivo* outcomes.

**Fig. 5:**
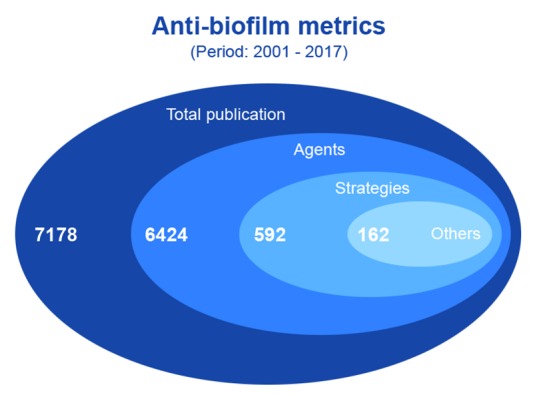
publication metrics in the anti-biofilm field. The search was performed in the PubMed database (www.pubmed.com) on April 1st, 2018, using the keywords “anti-biofilm”, “anti-biofilm agents”, and “anti-biofilm strategies”, and only papers published in English containing these words were selected for analysis. The graphic shows the number of publications in these biofilm arenas during the period 2001-2017, which is the most representative period for publications on biofilms (Fig. 4).

*Conclusions and perspectives* - The authors sincerely hope that the present mini-review arouses enthusiasm and scientific curiosity in students, teachers, and researchers worldwide since the intriguing and unique biofilm lifestyle of several microorganisms continues to be a challenge for both basic and applied research areas in clinical, industrial, and environmental fields across the academic and industry platforms. Without a doubt, due to the high level of tolerance/resistance shown by mature biofilms, it becomes a tremendously arduous task, many times impossible, to manage and eradicate biofilm-associated disease solely by conventional antimicrobial chemotherapy. Thus, we need to intensify our studies on microbial biofilms with the aim of deciphering and defeating this complex and robust social cooperation. With this perspective in mind, it is reasonable to expect that the combined herculean efforts in anti-biofilm research will lead to substantial improvements in both pharmaceuticals and medical practices.
